# Inflammation, immunity, and invasion: the pivotal role of cytokine storms in cervical carcinogenesis

**DOI:** 10.1097/MS9.0000000000004352

**Published:** 2025-11-17

**Authors:** Emmanuel Ifeanyi Obeagu

**Affiliations:** Department of Biomedical and Laboratory Science, Africa University, Mutare, Zimbabwe

**Keywords:** cervical cancer, cytokine storm, HPV, immunity, tumor microenvironment

## Abstract

Cervical cancer remains one of the leading causes of cancer-related deaths among women globally, with inflammation playing a significant role in its progression. The phenomenon of cytokine storm, characterized by an excessive and uncontrolled release of pro-inflammatory cytokines, has been identified as a critical factor driving the pathogenesis of cervical cancer. Cytokines such as interleukin-6 (IL-6), tumor necrosis factor-alpha (TNF-α), IL-1β, and IL-8 are implicated in enhancing tumor growth, immune evasion, and metastasis. This review aims to explore the mechanisms underlying cytokine storm in cervical cancer and its contribution to tumor progression, focusing on the role of inflammation in altering the tumor microenvironment and promoting metastatic spread. The cytokine storm in cervical cancer induces a series of molecular and cellular responses, including the activation of key signaling pathways such as Janus kinase/signal transducer and activator of transcription (JAK/STAT), nuclear factor-kappa B (NF-κB), and mitogen-activated protein kinase (MAPK), that drive tumor cell survival, proliferation, and invasiveness. The excessive production of these inflammatory cytokines alters the immune landscape, contributing to immune suppression and promoting an environment conducive to cancer cell survival. This dysregulated immune response not only enhances tumor aggressiveness but also renders the tumor more resistant to conventional therapies, posing significant challenges for treatment.

## Introduction

Cervical cancer is one of the most prevalent cancers among women worldwide, ranking as the fourth most common cancer globally, with an estimated 600 000 new cases and 340 000 deaths annually. The primary cause of cervical cancer is persistent infection with high-risk types of human papillomavirus (HPV), particularly HPV-16 and HPV-18. While HPV infection is necessary for the development of cervical cancer, it is not sufficient by itself, as additional factors such as genetic mutations, immune dysregulation, and chronic inflammation contribute significantly to disease progression. Among these factors, the role of inflammation in cervical cancer has gained significant attention in recent years due to its potential impact on tumor progression, metastasis, and therapeutic resistance^[1–4]^. The immune system plays a crucial role in protecting the body against malignancy; but when dysregulated, it can inadvertently contribute to cancer progression. In cervical cancer, chronic inflammation is a hallmark feature of the disease and is linked to a poor prognosis. One of the most devastating manifestations of chronic inflammation is the development of a cytokine storm, a condition characterized by the uncontrolled and excessive release of pro-inflammatory cytokines. These cytokines, such as interleukin-6 (IL-6), tumor necrosis factor-alpha (TNF-α), IL-1β, and IL-8, are typically involved in immune response and tissue repair; but in the context of cervical cancer, their dysregulated production leads to tumor promotion, immune suppression, and the establishment of a tumor microenvironment (TME) that favors tumor survival and metastasis^[5,6]^. Cytokine storms in cervical cancer are often triggered by the persistent viral infection, as HPV can directly interact with the host immune system and induce the release of inflammatory cytokines. The infiltration of immune cells, such as macrophages, neutrophils, and T lymphocytes, into the tumor site is driven by these cytokines, resulting in the TME becoming increasingly pro-inflammatory. This chronic inflammatory state supports cancer cell survival by enhancing angiogenesis, promoting epithelial-to-mesenchymal transition (EMT), and facilitating the immune escape of tumor cells. Furthermore, cytokine-induced immune suppression can result in the recruitment of regulatory T cells (Tregs) and myeloid-derived suppressor cells (MDSCs), which actively inhibit anti-tumor immune responses and promote tumor progression^[7–9]^.

**HIGHLIGHTS**

- Cytokine dysregulation: excessive inflammatory cytokines fuel tumor progression, creating a pro-tumor microenvironment.
- Angiogenesis boost: elevated vascular endothelial growth factor and interleukin-6 promote abnormal blood vessel formation, aiding cancer spread.
- Immune suppression: chronic inflammation depletes T-cell function, allowing tumor immune evasion.
- Epithelial-mesenchymal transition (EMT): pro-inflammatory cytokines trigger EMT, enhancing metastasis.
- Therapeutic challenges: targeting cytokine storms remains complex due to their dual roles in immunity and cancer progression.

A cytokine storm refers to an excessive and uncontrolled release of pro-inflammatory cytokines – such as IL-6, IL-1β, and TNF-α – resulting in sustained inflammation and immune dysregulation. In the context of cervical carcinogenesis, persistent high-risk HPV infection can initiate and amplify this response, creating a pro-tumorigenic environment that promotes immune evasion, tissue damage, and malignant transformation. The cytokine storm in cervical cancer also plays a significant role in promoting metastasis, which is the leading cause of cancer-related mortality. Inflammation-induced cytokine release drives changes in the tumor vasculature, enhancing the formation of new blood vessels to supply oxygen and nutrients to the growing tumor. Additionally, inflammatory cytokines like IL-6 and IL-8 have been shown to promote the migration and invasion of cancer cells through angiogenesis and by stimulating the production of matrix metalloproteinases (MMPs), which degrade the extracellular matrix (ECM) and facilitate cancer cell invasion into surrounding tissues and distant organs. In this way, cytokine storm not only promotes local tumor growth but also contributes to the spread of cancer to distant sites, worsening patient prognosis^[10–12]^. Furthermore, the cytokine storm plays a critical role in immune evasion, a hallmark of cervical cancer progression. Under normal circumstances, the immune system is able to recognize and eliminate tumor cells. However, in cervical cancer, the persistent inflammatory environment driven by cytokine storm can suppress the activation of cytotoxic immune cells, such as CD8+ T cells, and enhance the activity of immune-suppressive cells like Tregs and MDSCs. This results in a dysfunctional immune response that fails to effectively target and eliminate the tumor. In addition, cytokines such as IL-10 and TGF-β contribute to immune suppression by dampening the activity of pro-inflammatory cytokines and promoting a more immunosuppressive TME, further hindering the body’s ability to mount an anti-tumor immune response^[13,14]^. Given the critical role of cytokine storm in cervical cancer progression, targeting the inflammatory pathways that drive this process presents a promising therapeutic strategy. Various approaches have been explored, including the use of cytokine inhibitors, immune checkpoint inhibitors, and targeted therapies that aim to disrupt the signaling pathways responsible for the excessive production of inflammatory cytokines. For example, IL-6 inhibitors, such as tocilizumab, have shown efficacy in reducing cytokine-induced inflammation and improving outcomes in certain cancers. Immune checkpoint inhibitors, which target the PD-1/PD-L1 and CTLA-4 pathways, aim to reactivate the immune system and restore anti-tumor immune responses, counteracting the immune suppression caused by cytokine storm. These therapies hold significant potential for improving the effectiveness of existing cervical cancer treatments, particularly in patients with advanced disease or those who are refractory to conventional therapies[15].

## Aim

The aim of this review is to explore the mechanisms underlying the cytokine storm in cervical cancer, its impact on tumor progression and metastasis, and to critically evaluate the therapeutic strategies designed to target and mitigate this inflammatory response.

## Methodology

To ensure reproducibility and methodological rigor, a systematic and transparent approach was applied in the preparation of this narrative review. Literature searches were conducted across multiple electronic databases, including PubMed/MEDLINE, Embase, Scopus, Web of Science, and ClinicalTrials.gov, with the final search completed on 2010-2025. Search terms combined keywords and MeSH headings related to cervical cancer, HPV, cytokine storms, immune dysregulation, TME, and therapeutic interventions.

### Study selection and inclusion criteria

Articles were included if they met the following criteria: (1) published in peer-reviewed journals, (2) written in English, (3) reported mechanistic, clinical, or translational findings relevant to cytokine-mediated immune dysregulation and cervical carcinogenesis, and (4) provided original data or comprehensive reviews. Both *in vitro* and *in vivo* experimental studies, clinical trials, and observational human studies were considered. Exclusion criteria included non-English articles, studies lacking sufficient methodological detail, and grey literature without peer review. The rationale for excluding non-English studies and grey literature is provided in the Limitations section; these measures were applied to ensure data quality and reproducibility.

### Screening and data extraction

Two independent reviewers screened titles and abstracts for relevance, followed by full-text review. Discrepancies were resolved by discussion and consensus. Key data extracted included study design, sample size, HPV genotype or viral load, cytokine profiles, immune cell phenotypes, intervention type, outcomes, and reported adverse events where applicable. Quantitative data were standardized to include absolute counts, percentages, and 95% confidence intervals when reported in the primary sources.

### PRISMA flowchart

**Figure 1.**
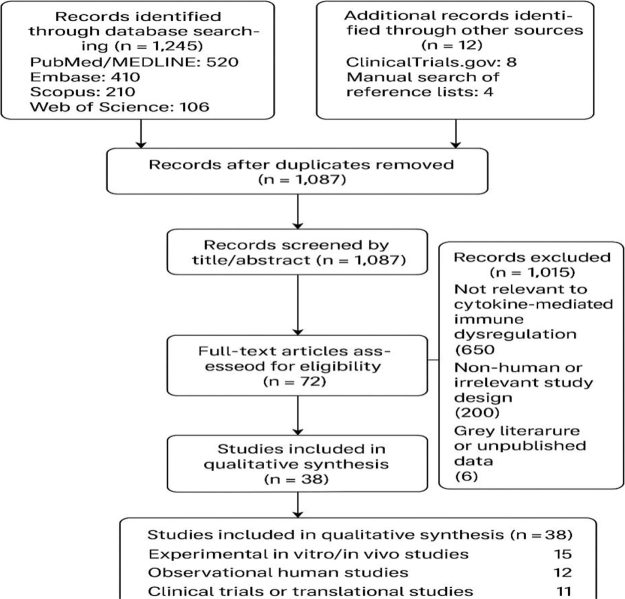
PRISMA flow chart.

The literature selection process is summarized in Figure 1, which outlines the number of records identified, screened, excluded (with reasons), and included in the narrative synthesis. This approach enhances transparency and allows readers to replicate the search strategy and verify inclusion decisions.

### Data synthesis

Studies were synthesized qualitatively to provide a cohesive narrative on cytokine-driven immune dysregulation, tumor invasion, and emerging therapeutic strategies. Where possible, data reporting was standardized to ensure consistency across studies and facilitate interpretation of mechanistic, clinical, and translational evidence.

This structured and transparent methodology ensures that the review is reproducible, reliable, and systematically grounded in the available evidence, while acknowledging the limitations inherent to language restrictions and exclusion of non–peer-reviewed sources.

## Mechanisms of cytokine storm in cervical cancer

Cervical cancer is closely linked to persistent inflammation, with cytokine storm playing a pivotal role in disease progression. In the case of cervical cancer, this inflammatory response is often exacerbated by persistent HPV infection, which can activate several immune and non-immune cells within the TME. The cytokine storm in cervical cancer is not only involved in promoting tumor growth but also plays a significant role in immune evasion, angiogenesis, metastasis, and therapy resistance^[12,16]^. One of the primary mechanisms of cytokine storm in cervical cancer is the excessive release of cytokines such as IL-6, TNF-α, IL-1β, and IL-8. These cytokines are often upregulated as a result of HPV infection, a key etiological factor in cervical carcinogenesis. HPV, particularly high-risk types such as HPV-16 and HPV-18, produces viral proteins that interact with host cell machinery, thereby inducing inflammatory responses. HPV infection leads to the activation of several signaling pathways, including the nuclear factor-kappa B (NF-κB), Janus kinase/signal transducer and activator of transcription (JAK/STAT), and mitogen-activated protein kinase (MAPK) pathways, which are responsible for the production of these inflammatory cytokines. The NF-κB pathway, in particular, is a critical regulator of inflammation and immune responses. In cervical cancer, the persistent activation of NF-κB leads to the chronic production of cytokines that exacerbate tumor progression and promote immune suppression[15].

In the TME, cytokines such as IL-6 and IL-8 have been found to enhance several processes that are central to cervical cancer progression, such as angiogenesis, EMT, and cell migration. IL-6, in particular, is one of the most studied cytokines in cervical cancer, where it promotes the growth and survival of tumor cells. IL-6 can also activate the JAK/STAT3 signaling pathway, which in turn promotes the expression of genes that are involved in immune evasion, metastasis, and tumor cell survival. The dysregulation of this pathway leads to an environment that not only sustains tumor growth but also enhances tumor cell migration and invasion, essential processes for metastasis. Elevated levels of IL-8, another key cytokine, contribute to neovascularization, a critical component of tumor growth and metastasis. IL-8 induces endothelial cell proliferation and migration, promoting angiogenesis, which provides tumors with the oxygen and nutrients necessary for further expansion and spread^[17,18]^. Cytokine-induced immune dysregulation also plays a significant role in cervical cancer. The excessive production of pro-inflammatory cytokines suppresses the activity of cytotoxic immune cells, including CD8+ T cells, which are essential for recognizing and eliminating cancer cells. In the context of cytokine storm, immune suppressive cells such as Tregs and MDSCs are recruited to the tumor site, where they further inhibit anti-tumor immunity. The recruitment of these cells is primarily driven by cytokines like IL-10 and TGF-β, which contribute to the establishment of an immunosuppressive microenvironment. This not only hampers the body’s ability to mount an effective immune response but also promotes tumor immune evasion, a critical step in cancer progression^[9,19]^.

The cytokine storm in cervical cancer also alters the composition and function of the ECM, which is crucial for tumor invasion and metastasis. Cytokines such as TNF-α and IL-1β stimulate the production of MMPs, enzymes that degrade the ECM and facilitate the migration of cancer cells. This process, known as EMT, is a key feature of metastasis, enabling tumor cells to acquire migratory and invasive properties. In this manner, cytokine-induced EMT not only promotes local tumor growth but also allows for the spread of cancer cells to distant organs, worsening patient prognosis. Furthermore, cytokines in the TME can induce chemotaxis, recruiting other immune and non-immune cells that further contribute to the growth and spread of the tumor^[20,21]^. One of the significant challenges in treating cervical cancer is the development of therapy resistance, which is closely linked to the presence of a cytokine storm. The cytokine storm contributes to therapy resistance by promoting the survival of tumor cells despite treatment. For example, IL-6 and TNF-α have been shown to protect tumor cells from the cytotoxic effects of chemotherapy and radiation by activating survival pathways such as AKT and MAPK. This is often referred to as cytokine-mediated chemoresistance. Furthermore, cytokine-induced immune suppression can impair the effectiveness of immunotherapies, including immune checkpoint inhibitors, by inhibiting the activity of effector immune cells and enhancing the recruitment of immune-suppressive cells. This highlights the need for novel therapeutic approaches targeting cytokine signaling pathways to overcome resistance and improve treatment outcomes in cervical cancer^[22,23]^. Another important aspect of the cytokine storm in cervical cancer is its contribution to the tumor-associated inflammation. Chronic inflammation, in general, fosters an environment that is conducive to genetic mutations, cellular stress, and subsequent malignant transformation. In the case of cervical cancer, the presence of HPV infection perpetuates this inflammatory cycle, leading to a genetically unstable environment that promotes cancer development. The chronic inflammatory response also increases the risk of angiogenesis, cell survival, and genomic instability, all of which are central to tumor initiation and progression. Consequently, targeting the inflammation-driven mechanisms of cytokine storm is a promising strategy for preventing tumor progression and metastasis in cervical cancer[24].

High-risk HPV infection, particularly with types 16 and 18, plays a central role in cervical carcinogenesis through the activity of its viral oncoproteins E6 and E7. These proteins interfere with host cellular regulatory mechanisms not only by degrading tumor suppressors p53 and pRb but also by manipulating key inflammatory signaling pathways. E6 has been shown to activate the NF-κB pathway, leading to increased transcription of pro-inflammatory cytokines such as IL-6 and TNF-α, which contribute to a chronic inflammatory microenvironment. Concurrently, E7 promotes activation of the STAT3, a transcription factor involved in immune suppression and tumor promotion. Activation of STAT3 upregulates genes involved in cytokine production, angiogenesis, and EMT, thereby facilitating immune evasion and tumor invasion. Together, E6- and E7-mediated dysregulation of NF-κB and STAT3 not only drives persistent inflammation but also enables the virus to subvert host immunity, creating favorable conditions for tumor initiation and progression (Table 1)^[23,24]^.

**Table 1:** Crosstalk between JAK/STAT, NF-κB, and MAPK pathways in cytokine-driven cervical cancer progression

Pathway	Key activating cytokines	Major downstream effects	Interaction with other pathways	Relevance to cervical cancer
JAK/STAT	IL-6, IL-10, IFN-γ	Promotes STAT3-mediated transcription of genes involved in proliferation, immune suppression, angiogenesis	Activates NF-κB and MAPK via IL-6 feedback loop; cross-talk with HPV E7	Enhances tumor cell survival, immune evasion, and chronic inflammation
NF-κB	TNF-α, IL-1β, IL-6	Induces expression of pro-inflammatory genes, anti-apoptotic factors, cytokines	Upregulates IL-6 → activates JAK/STAT; activated by HPV E6	Sustains inflammation, inhibits apoptosis, promotes invasion
MAPK (ERK, JNK, p38)	IL-1, TNF-α, growth factors	Regulates cell proliferation, differentiation, and stress responses	Can be co-activated with NF-κB by cytokines; modulates STAT3 activity	Facilitates tumor growth, EMT, and resistance to therapy

EMT, epithelial-to-mesenchymal transition; ERK, extracellular signal-regulated kinase; HPV, human papillomavirus; IL, interleukin; IFN, interferon; JAK/STAT, Janus kinase/signal transducer and activator of transcription; JNK, c-Jun N-terminal kinase; MAPK, mitogen-activated protein kinase; NF-κB, nuclear factor-kappa B.

## Immune dysregulation and tumor invasion

Tumor progression in cervical cancer is closely intertwined with alterations in the host immune system. Persistent high-risk HPV infection initiates a chronic inflammatory response, but over time, this initially protective immunity becomes dysregulated, creating a permissive environment for tumor growth and invasion. A hallmark of this process is the imbalance between pro-inflammatory and immunosuppressive pathways, which allows tumor cells to evade immune surveillance while simultaneously promoting tissue remodeling and metastatic potential[25]. One of the key mechanisms driving immune dysregulation is the sustained production of pro-inflammatory cytokines, including IL-6, IL-8, TNF-α, and TGF-β. While these cytokines initially aim to contain viral infection, chronic exposure results in several pro-tumor effects. IL-6, through the activation of the JAK/STAT3 pathway, promotes EMT, enhances proliferation, and facilitates survival of dysplastic cells under stress conditions. IL-8 contributes to neovascularization and recruits neutrophils that release proteolytic enzymes, further destabilizing the ECM and enabling tumor cell migration. TGF-β, although tumor-suppressive in early stages, acquires pro-invasive properties in established lesions, driving EMT and facilitating immune evasion. Collectively, these cytokine-mediated events not only provide growth advantages but also sculpt a TME that is immunosuppressive and structurally conducive to invasion[26].

Immune cell composition within the TME is profoundly altered. Regulatory T cells (Tregs) and MDSCs expand in response to persistent inflammatory signaling, suppressing cytotoxic T lymphocyte (CTL) activity and dampening effective anti-tumor responses. Tumor-associated macrophages (TAMs) are skewed toward an M2 phenotype, secreting angiogenic factors and MMPs that degrade ECM components, facilitating tumor dissemination. Chronic cytokine exposure also promotes immune checkpoint expression, further inhibiting T-cell activation and allowing transformed cells to persist unchecked[27]. The interplay between cytokine-driven inflammation and immune suppression accelerates invasion by creating physical and molecular pathways for tumor spread. Proteolytic degradation of the ECM by MMPs and other enzymes provides channels for malignant cells to penetrate neighboring tissues. Simultaneously, the release of angiogenic factors establishes new vasculature, supplying nutrients and enabling systemic dissemination. These events underscore how chronic immune dysregulation transforms the TME into a pro-metastatic niche, effectively linking persistent HPV infection, aberrant cytokine signaling, and tumor invasiveness[21]. Modulating cytokine activity, reprogramming suppressive immune cells, and normalizing the TME represent promising strategies to halt progression and reduce metastatic potential. In this context, the study of immune-tumor interactions remains central to developing interventions that restore anti-tumor immunity while limiting the deleterious effects of chronic inflammation[28].

## Context-dependent roles of key cytokines in cervical cancer: IL-6 and TNF-α

**Figure 2.**
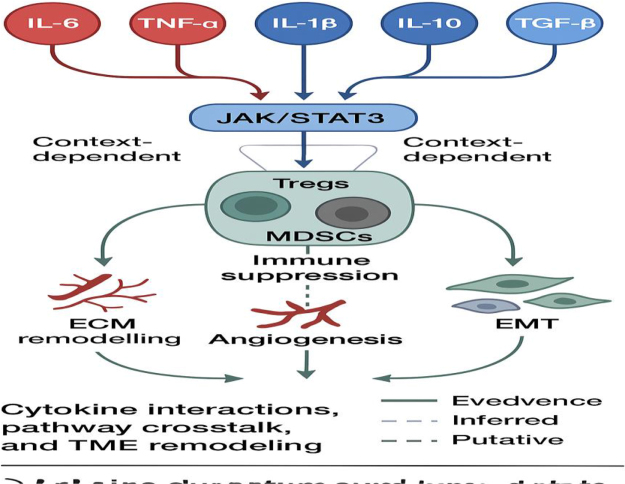
Schematic of cytokine interactions, pathway crosstalk, and TME remodeling.

Cytokines such as IL-6 and TNF-α are pivotal mediators of the inflammatory microenvironment in cervical cancer, yet their roles are complex and context-dependent. Both cytokines can promote tumor progression by fostering chronic inflammation, immune suppression, and enhancing processes like angiogenesis and EMT. For instance, IL-6-driven activation of the JAK/STAT3 pathway supports cervical tumor cell proliferation, survival, and immune evasion, while TNF-α can activate NF-κB signaling, leading to the transcription of genes that inhibit apoptosis and promote invasion[25]. Conversely, under certain conditions, these cytokines may exert anti-tumor effects. TNF-α, initially identified for its tumor necrosis-inducing properties, can trigger apoptotic pathways in tumor cells and activate cytotoxic immune responses, especially in early disease stages or acute inflammatory settings. Similarly, IL-6 may enhance anti-tumor immunity by stimulating immune cell differentiation and activating acute-phase responses. The dichotomous nature of these cytokines is influenced by multiple factors, including the cytokine concentration, cellular source, TME composition, and stage of disease progression^[26,27]^. In cervical cancer, persistent HPV infection shifts the balance towards chronic low-grade inflammation where IL-6 and TNF-α predominantly support tumorigenesis. However, transient or localized cytokine bursts may still contribute to immune-mediated tumor clearance. Understanding these nuanced roles is critical for designing targeted therapies, as blocking these cytokines indiscriminately may disrupt beneficial anti-tumor immune functions. Therefore, therapeutic strategies must consider the temporal and spatial dynamics of cytokine signaling within the TME to effectively harness their dual potential (Fig. 2)^[21,28]^.

## Therapeutic strategies

The intricate interplay between cytokine-mediated inflammation, immune dysregulation, and cervical tumor progression has unveiled several therapeutic opportunities. Targeting these pathways offers the potential not only to curb tumor growth but also to restore effective anti-tumor immunity. Current strategies encompass cytokine-targeted interventions, immune checkpoint modulation, combination therapies with conventional treatments, and biomarker-guided approaches[23]. Pro-inflammatory cytokines such as IL-6 and IL-8 are central drivers of tumor progression, angiogenesis, and epithelial–EMT. Agents targeting these cytokines or their downstream pathways are under investigation. For instance, IL-6 blockade via monoclonal antibodies or JAK/STAT3 inhibitors has demonstrated early promise in solid tumors, including cervical cancer, by reducing proliferation, reversing EMT, and sensitizing tumor cells to chemotherapy and radiotherapy. Similarly, strategies targeting IL-8 and vascular endothelial growth factor (VEGF) aim to reduce angiogenesis and limit tumor dissemination. Clinical trials report objective response rates in the range of 20–25% in early-phase studies, with adverse effects generally manageable; however, confidence intervals and longer-term outcomes are still emerging^[29,30]^.

Dysregulated cytokine signaling contributes to the expansion of immunosuppressive populations such as Tregs and MDSCs and upregulates inhibitory checkpoints (PD-1, PD-L1, CTLA-4). Immune checkpoint inhibitors have thus emerged as a complementary approach to restore cytotoxic T-cell activity. Pembrolizumab, for example, is approved for advanced or recurrent cervical cancer, demonstrating durable responses in a subset of patients. Integrating cytokine-targeted therapy with checkpoint blockade may enhance anti-tumor immunity by mitigating the immunosuppressive microenvironment[31]. Combining cytokine inhibitors with conventional chemotherapy or radiotherapy holds particular promise, as inflammation-driven resistance mechanisms can limit treatment efficacy. Rational sequencing – whether concurrent or sequential administration – can optimize synergistic effects while minimizing toxicity. However, combination therapy carries risks, including heightened infection susceptibility, impaired tissue repair, and potential exacerbation of treatment-related side effects. Careful patient selection, biomarker-guided stratification, and vigilant monitoring are essential to mitigate these risks[32].

Incorporating serum or tissue biomarkers, such as IL-6, VEGF, and cytokine profiles, allows stratification of patients likely to benefit from targeted interventions. Dynamic monitoring of these markers during therapy may also inform dose adjustments and early detection of resistance or adverse effects. Personalized approaches hold the promise of enhancing efficacy while minimizing unnecessary exposure to potentially toxic agents[33]. Emerging strategies include combining cytokine-targeted therapies with novel immunotherapies, adoptive T-cell therapies, and therapeutic vaccines, particularly in the context of persistent high-risk HPV infection. Additionally, ongoing research seeks to delineate cytokine dynamics across HPV subtypes, providing opportunities to tailor therapy based on viral genotype and tumor immunophenotype.

## Future directions

Despite substantial progress in understanding the role of cytokine storms in cervical carcinogenesis, key knowledge gaps remain that warrant focused investigation.

## Cytokine biomarkers for stratification and monitoring

Emerging evidence supports the potential of circulating cytokines, particularly IL-6 and VEGF, as biomarkers for patient stratification and disease monitoring. Elevated serum IL-6 has been correlated with tumor burden, immune dysregulation, and poor prognosis, while VEGF levels reflect angiogenic activity within the TME. To translate these findings into clinical practice, several critical steps are required: standardization of assay protocols to ensure reproducibility across laboratories, establishment of reference ranges in healthy versus at-risk populations, and prospective validation in longitudinal cohorts to determine predictive value for treatment response and recurrence[30].

## HPV genotype-specific immune responses

Cytokine dynamics may differ according to HPV genotype. Available, albeit limited, evidence suggests that infections with HPV-16 and HPV-18 are associated with more pronounced and sustained cytokine elevations compared to other high-risk types, potentially contributing to higher persistence and malignancy risk. To elucidate these differences, longitudinal, genotype-stratified studies are needed. Such studies should track cytokine profiles from initial infection through lesion progression, enabling identification of genotype-specific immune signatures that could inform personalized risk assessment, vaccine strategies, and targeted immunomodulatory therapies[31].

## Integration into clinical practice and translational research

Future research should focus on integrating cytokine biomarkers and HPV genotype data into risk-adapted clinical algorithms. Additionally, mechanistic studies investigating the interplay between viral oncogenes, cytokine networks, and the TME may identify novel therapeutic targets. Finally, interdisciplinary collaboration across immunology, virology, and oncology will be essential to translate these insights into actionable clinical interventions that improve early detection, prognostication, and patient outcomes^[32,33]^.

## Conclusion

Cytokine storm plays a pivotal role in the progression and metastasis of cervical cancer, contributing to a pro-inflammatory environment that supports tumor growth, immune evasion, and the spread of cancer cells. The mechanisms underlying cytokine storm in cervical cancer are complex, involving the dysregulation of immune responses, upregulation of pro-inflammatory cytokines, and the activation of key signaling pathways that promote metastasis. This hyper-inflammatory state exacerbates the challenges of treatment, making metastatic cervical cancer particularly difficult to manage. Therapeutic strategies targeting cytokine storm in cervical cancer have emerged as promising approaches to mitigate its devastating effects. By neutralizing specific pro-inflammatory cytokines, inhibiting key signaling pathways, and utilizing immune-modulating agents, these therapies aim to restore immune homeostasis and reduce the inflammatory burden that supports tumor progression. Additionally, advanced strategies such as nanomedicines and gene therapy hold significant potential to provide more targeted and personalized treatments, further improving the management of cervical cancer.

## Data Availability

Not applicable as this a perspective.

## Research registration unique identifying number (UIN)

Not applicable as this a perspective.
